# Supplementary material for: Application of Thyroid Hormones in Women’s Hair for the Non-Invasive Prediction of Graves’ Disease
Source: Biomolecules. 2025 Feb 28;15(3):353. doi: 10.3390/biom15030353 (PMC11940391; doi:10.3390/biom15030353)
Supplement: Supplementary file 1 [file biomolecules-15-00353-s001.zip › biomolecules-3452542-supplementary.pdf]

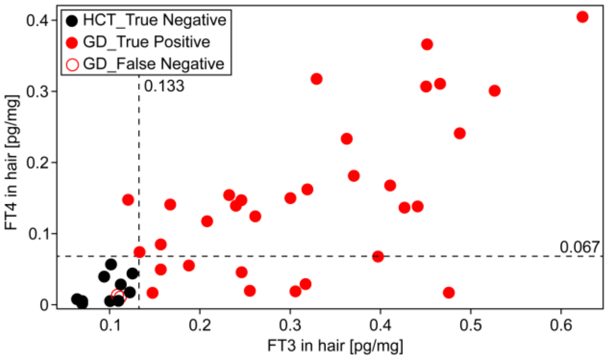


| FT3 & FT4 | | Actual | | Cut off<br>0.133 pg/mg (FT3)<br>0.067 pg/mg (FT4) |
|---|---|---|---|---|
| | | Positive | Negative | |
| Predicted | Positive | 32 | 0 | PPV<br>100% |
| | Negative | 2 | 10 | NPV<br>83.3% |
| Youden index | | Sensitivity<br>94.1% | Specificity<br>100% | Accuracy<br>95.5% |

**Figure S1.** Predictive determination of GD using two thyroid hormone components in hair. Two-axis plot of the amounts of FT3 and FT4 in the hair of HCT volunteers (true negative: filled black circle) and GD patients (true positive: filled red circle; false negative: empty red circle). The cut-off values (dashed line) are 0.133 pg/mg and 0.067 pg/mg for FT3 and FT4, respectively (sensitivity: 94.1%; specificity: 100%; PPV: 100%; NPV: 83.3%).
